# Reliability and Validity of Electronic Patient-Reported Outcomes Using the Smartphone App AllerSearch for Hay Fever: Prospective Observational Study

**DOI:** 10.2196/38475

**Published:** 2022-08-23

**Authors:** Yasutsugu Akasaki, Takenori Inomata, Jaemyoung Sung, Yuichi Okumura, Kenta Fujio, Maria Miura, Kunihiko Hirosawa, Masao Iwagami, Masahiro Nakamura, Nobuyuki Ebihara, Masahiro Nakamura, Takuma Ide, Ken Nagino, Akira Murakami

**Affiliations:** 1 Department of Ophthalmology Juntendo University Graduate School of Medicine Tokyo Japan; 2 Department of Digital Medicine Juntendo University Graduate School of Medicine Tokyo Japan; 3 Department of Hospital Administration Juntendo University Graduate School of Medicine Tokyo Japan; 4 Department of Health Services Research Faculty of Medicine University of Tsukuba Ibaraki Japan; 5 Precision Health, Department of Bioengineering Graduate School of Engineering The University of Tokyo Tokyo Japan; 6 Department of Ophthalmology Urayasu Hospital Juntendo University Chiba Japan; 7 Department of Otorhinolaryngology, Head and Neck Surgery Juntendo University Faculty of Medicine Tokyo Japan

**Keywords:** hay fever, AllerSearch, smartphone app, mobile health, mHealth, patient-reported outcome, reliability, validity, Japanese Allergic Conjunctival Disease Standard Quality of Life Questionnaire, JACQLQ, questionnaire, allergic conjunctivitis

## Abstract

**Background:**

Hay fever is a highly prevalent, heterogenous, and multifactorial disease. Patients may benefit from longitudinal assessments using mobile health (mHealth) principles. We have previously attempted to establish an effective mHealth platform for patients with hay fever through AllerSearch, our in-house smartphone app that assesses electronic patient-reported outcomes through a questionnaire on hay fever and provides evidence-based advice. To be used by the public, an investigation on its reliability and validity is necessary.

**Objective:**

The aim of this paper is to assess the reliability and validity of subjective symptom data on hay fever collected through our app, AllerSearch.

**Methods:**

This study used a prospective observational design. The participants were patients aged ≥20 years recruited from a single university hospital between June 2, 2021, and January 26, 2022. We excluded patients who could not use smartphones as well as those with incomplete data records and outlier data. All participants answered the Japanese Allergic Conjunctival Disease Standard Quality of Life Questionnaire (JACQLQ), first in the paper-and-pencil format and subsequently on AllerSearch on the same day. The JACQLQ comprises the following three domains: Domain I, with 9 items on ocular or nasal symptoms; Domain II, with 17 items on daily activity and psychological well-being; and Domain III, with 3 items on overall condition by face score. The concordance rate of each domain between the 2 platforms was calculated. The internal consistency of Domains I and II of the 2 platforms was assessed using Cronbach alpha coefficients, the concurrent validity of Domains I and II was assessed by calculating Pearson correlation coefficients, and the mean differences between the 2 platforms were assessed using Bland-Altman analysis.

**Results:**

In total, 22 participants were recruited; the data of 20 (91%) participants were analyzed. The average age was 65.4 (SD 12.8) years, and 80% (16/20) of the participants were women. The concordance rate of Domains I, II, and III between the paper-based and app-based JACQLQ was 0.78, 0.85, and 0.90, respectively. The internal consistency of Domains I and II between the 2 platforms was satisfactory (Cronbach alpha of .964 and .919, respectively). Pearson correlation analysis yielded a significant positive correlation between Domains I and II across the 2 platforms (*r*=0.920 and *r*=0.968, respectively). The mean difference in Domains I and II between the 2 platforms was 3.35 units (95% limits of agreement: –6.51 to 13.2).

**Conclusions:**

Our findings indicate that AllerSearch is a valid and reliable tool for the collection of electronic patient-reported outcomes to assess hay fever, contributing to the advantages of the mHealth platform.

## Introduction

Hay fever is currently believed to be the most common immunologic and allergic disease worldwide, with reports of nearly 30 million cases in the United States and Japan [1-3]. Hay fever symptoms can be chronic and therefore life altering, leading to a decrease in individuals’ quality of life and work productivity [3]. This systemic illness targets multiple organs, most commonly manifesting as allergic rhinitis, conjunctivitis, and dermatitis [4,5]. The disease appears to evolve, changing its presentation with varying onsets, levels of severity, and responses to treatment based on the individual [3,4]. Therefore, a deeper understanding of the underlying pathophysiology and establishing an effective strategy to comprehensively assess changing symptoms become imperative to provide tailored, longitudinal care and to improve patients’ quality of life [4-7].

Recent findings have increasingly confirmed the advantages of adopting patient-reported outcomes (PROs), which are clinical data grounded in patients’ own subjective experiences that are not readily captured by routine medical evaluations [8,9]. With the recent advancements in mobile health (mHealth), a medical discipline centered around health care and support through advanced mobile devices such as smartphones, the electronic adaptation of PROs (ePROs) has been garnering attention as a novel data accrual option for clinical researchers [4,5,10-12].

We have previously taken advantage of the novel mHealth platform and conducted studies through our in-house hay fever smartphone app, AllerSearch, released in February 2018 [4,5]. The app successfully gathered comprehensive medical data related to hay fever without interrupting users’ daily lives. By using data collected through AllerSearch, we were able to elucidate various risk factors that could exacerbate the disease, and we stratified the disease into subgroups based on collective symptoms and individual factors [4,5]. In our efforts to implement ePROs via AllerSearch, the app was equipped with features to administer hay fever symptom–related questionnaires, such as the Japanese Allergic Conjunctival Disease Standard Quality of Life Questionnaire (JACQLQ) [4,5,13,14]. Given the ongoing pandemic and the anticipated postpandemic era, the demand for longitudinal, nonintrusive health care continues to increase, and mHealth appears to hold the key to addressing this need. To realize such nonintrusive care that can also engage the principles of participatory medicine through mHealth, a robust validation of mHealth-accrued clinical data on subjective symptoms and their quantification strategies is required.

Hence, we evaluated the reliability and validity of the subjective symptom data collected through our mHealth app by conducting a comparative study between paper-based and app-based versions of the JACQLQ to evaluate the applicability of AllerSearch as a novel clinical tool for assessing hay fever.

## Methods

### AllerSearch Smartphone App

AllerSearch was initially developed in Japan using Apple Inc’s open-source framework, ResearchKit [4,5], and released on Apple’s App Store on February 1, 2018, under a consignment contract with Juntendo University Graduate School of Medicine and InnoJin, Inc, both based in Tokyo, Japan. The Android version was released on August 26, 2020. The AllerSearch is freely available on the App Store and Google play.

### Design

This study employed a prospective observational design based on previously published validation studies of medical instruments [15,16].

### Ethical Considerations

Written informed consent was obtained from all participants prior to the commencement of the study. The study was approved by the Independent Ethics Committee of Juntendo University Graduate School of Medicine (approval number H20-0242-H01, November 6, 2020) and adheres to the tenets of the Declaration of Helsinki.

### Enrollment and Participants

The participants were patients aged ≥20 years, recruited between June 2, 2021, and January 26, 2022, from the Department of Ophthalmology, Juntendo University Hospital, Tokyo, Japan. We excluded patients who could not use smartphones as well as those with incomplete data records and outlier data.

All participants answered the paper-based JACQLQ at the outpatient service in the Department of Ophthalmology, Juntendo University Hospital. They subsequently answered the same questionnaire on an iOS version of AllerSearch (app-based JACQLQ) on the same day. AllerSearch had been preinstalled on the mobile phones provided for the purpose of this study. Our previous study contained the description of survey items in AllerSearch [4]. Briefly, participants provided electronic consent and basic information on demographics, medical history, lifestyle, hay fever status, and preventive behavior for hay fever. Subsequently, participants performed daily assessments of their conjunctiva and responded to a questionnaire on hay fever that included the JACQLQ and assessments of nasal symptoms, nonnasal symptoms, daily subjective symptoms, and work productivity.

### JACQLQ

The JACQLQ is a well-established metric that enables clinicians to comprehensively assess QOL among patients in the Japanese-speaking population who are affected by allergic conjunctival diseases [13]. The JACQLQ comprises the following three domains: Domain I with 9 items on ocular or nasal symptoms; Domain II with 17 items on daily activity and psychological well-being; and Domain III with 3 items on overall condition by face score. The questionnaire requires participants to rate each symptom on a 5-point Likert scale according to its severity, from “None” (0 points) to “Severe” (4 points). The total score (Domains I and II) for the scale and the total score of each domain was calculated as the sum of Domains I and II and the sum of items in each domain, respectively. Of note is that the default settings of the scale bar in AllerSearch, used to represent the 5-point scale, and the face score were both set to the lowest score, but users were able to adjust their ratings to higher scores as they deemed fit.

### Statistical Analysis

The sample size for the Cronbach alpha test was predetermined based on the formula by Bonett [17]. Using these settings—power=90%, significance level=5%, number of items (k)=26, value of Cronbach alpha at null hypothesis=.0, and expected value of Cronbach alpha=.7—the required sample size was calculated to be 17.08 (rounded up to 18) cases. Accounting for 20% dropouts owing to missing data or withdrawal of consent, the final sample size was 22 cases.

The median scores for the paper-based and app-based JACQLQ were compared using Wilcoxon matched-pairs signed-rank tests [18,19]. The concordance rate of each item and domain between the 2 platforms was calculated. The internal consistency of the app-based JACQLQ was assessed using Cronbach alpha coefficient, with an alpha score of >.70 considered acceptable [20]. Concurrent validity was assessed by calculating the correlations (Pearson coefficient) and mean differences (Bland-Altman analysis) [15,21].

Statistical analyses were performed using Stata/MP version 16.1 (Stata Corp) and GraphPad Prism version 9.1.2 (GraphPad Software). Statistical significance was set at *P*<.05.

### Patient and Public Involvement

Input on the AllerSearch survey questionnaire was obtained to produce a version that was agreed upon by a committee comprising allergy specialists, ophthalmologists, otolaryngologists, epidemiologists, and the patient and public involvement members [4,5].

## Results

### Participant Characteristics

In total, 22 participants were recruited for this study. Following the exclusion of an individual with incomplete data records and another with outlier data, the data of 20 (91%) participants were analyzed. Table 1 shows the participants’ characteristics. The average age was 65.4 (SD 12.8) years, and 80% (16/20) were female participants. The mean best-corrected visual acuity value for both eyes was –0.06 (SD 0.05). The mean intraocular pressure was 13.9 (SD 2.6) mmHg.

**Table 1:** Participants’ characteristics (N=20).

Characteristics			Values
Age (year), mean (SD)			65.4 (12.8)
**Gender, n (%)**			
	Female	16 (80)	
	Male	4 (20)	
BCVA^a^, logMAR (SD)			–0.04 (0.07)
IOP^b^, mmHg (SD)			13.9 (2.6)

^a^BCVA: best-corrected visual acuity.

^b^IOP: intraocular pressure.

### Scores and Concordance Rate of Paper-Based and App-Based JACQLQ

The median total score for Domains I and II was 6.5 (range: 1.75-13.25) for the paper-based JACQLQ and 4.5 (range: 1–8) for the app-based JACQLQ (*P*=.003). Table 2 shows each item’s median score and concordance rate for the paper-based and app-based JACQLQ. The individual total score of Domains I and II was significantly higher in the paper-based JACQLQ compared with the app-based JACQLQ. The concordance rates of each item, subscale, and domain were more than 70%.

**Table 2:** JACQLQ^a^ item scores and concordance rate between paper-based and app-based JACQLQ.

JACQLQ items				Paper-based JACQLQ, median (IQR)		App-based JACQLQ, median (IQR)		*P* value		Concordance rate (%)
**Domain I, 0-36**				3.5 (1.75-6)		2 (1-4.25)		.004		78
	**Eye symptoms, 0-20**			2 (1-4.25)		1.5 (0-3)		.01		82
		1. Itchy eyes	1 (0-1)		1 (0-1)		>.99		95	
		2. Foreign body sensation	1 (0-1)		0 (0-1)		.12		65	
		3. Red eyes	0 (0-1)		0 (0-0.25)		.13		80	
		4. Watery eyes	0 (0-0)		0 (0-0)		.50		90	
		5. Eye discharge	0 (0-1)		0 (0-0.25)		.13		80	
	**Nasal symptoms, 0-16**			1 (0-2.25)		0.5 (0-1)		.01		74
		6. Runny nose	0 (0-1)		0 (0-0.25)		.06		75	
		7. Sneezing	0.5 (0-1)		0 (0-0.25)		.03		70	
		8. Stuffy nose	0 (0-1)		0 (0-0)		.38		75	
		9. Itchy nose	0 (0-0.25)		0 (0-0)		.06		75	
**Domain II, 0-68**				2 (0-5.5)		1.5 (0-5.5)		.04		85
	**Daily activity, 0-44**			2 (0-4.25)		0.5 (0-2)		.002		85
		1. Obstacles to studying, working, and housework	0 (0-1)		0 (0-0)		.13		80	
		2. Poor mental concentration	0 (0-1)		0 (0-0)		.50		90	
		3. Decreased thinking ability	0 (0-0)		0 (0-0)		>.99		85	
		4. Impaired reading newspapers and other materials	0 (0-1)		0 (0-1)		.13		80	
		5. Poor memory	0 (0-1)		0 (0-0)		.50		90	
		6. Limitation of outdoor life such as sports and picnics	0 (0-0)		0 (0-0)		>.99		85	
		7. Limitation of going out	0 (0-0)		0 (0-0)		.38		80	
		8. Obstacles to socializing with people	0 (0-0)		0 (0-0)		.50		90	
		9. Interfering with conversations and telephone calls with others	0 (0-0)		0 (0-0)		>.99		90	
		10. Anxiety about people around you	0 (0-0)		0 (0-0)		>.99		95	
		11. Sleeping disorder	0.5 (0-1)		0 (0-1)		.03		70	
	**Psychological well-being, 0-24**			0 (0-1.25)		0 (0-2)		>.99		86
		12. Dullness	0 (0-0.25)		0 (0-0.25)		>.99		80	
		13. Fatigue	0 (0-0)		0 (0-1)		.75		85	
		14. Frustrated	0 (0-0)		0 (0-0)		.25		85	
		15. Irritable	0 (0-0)		0 (0-0)		>.99		95	
		16. Depressed	0 (0-0.25)		0 (0-0)		.63		80	
		17. Dissatisfaction with life	0 (0-0)		0 (0-0)		>.99		90	
Domain III, 0-4				1 (1-2)		1 (1-2)		>.99		90

^a^JACQLQ: Japanese Allergic Conjunctival Disease Standard Quality of Life Questionnaire.

### Reliability Between App-Based and Paper-Based JACQLQ

The internal consistency of the total, subscale, and domain scores between the paper-based and app-based JACQLQ is indicated in Table 3. Our results show satisfactory internal consistency for most questionnaire items (Cronbach alpha>.70), except for nasal symptoms in the app-based version (Cronbach alpha=.331).

**Table 3:** Reliability between the paper-based and app-based JACQLQ^a^.

JACQLQ		No. of items	Cronbach alpha	
			Paper-based	App-based
Domains I and II		26	.964	.919
**Domain I**		9	.897	.746
	Eye symptoms	5	.856	.788
	Nasal symptoms	4	.776	.331
**Domain** **II**		17	.953	.896
	Daily activity	11	.914	.915
	Psychological well-being	6	.937	.731

^a^JACQLQ: Japanese Allergic Conjunctival Disease Standard Quality of Life Questionnaire.

### Correlation Between App-Based and Paper-Based JACQLQ

**Figure 1:**
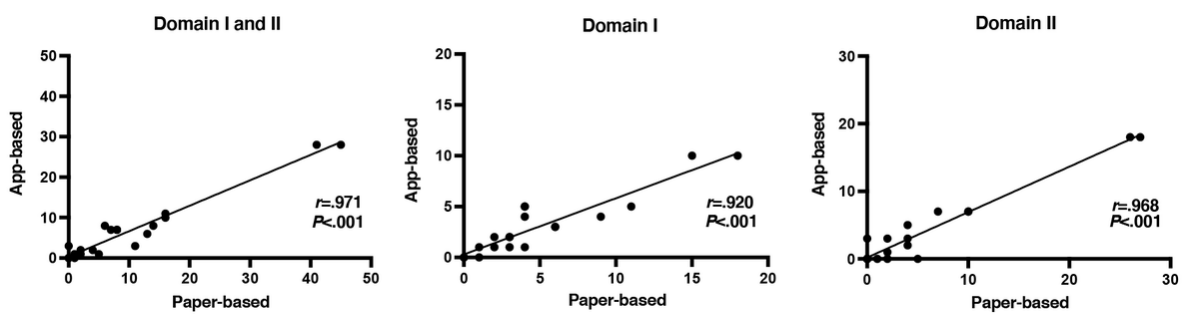
Correlation between the app-based and paper-based Japanese Allergic Conjunctival Disease Standard Quality of Life Questionnaires (JACQLQs). The correlation between the paper-based and app-based JACQLQs of Domains I and II, Domain I, and Domain II.

Figure 1 shows the correlation between the paper-based and app-based JACQLQ. There were significant positive correlations between the 2 measurements (Domains I and II: *r*=0.971, *P*<.001; Domain I: *r*=0.920, *P*<.001; and Domain II: *r*=0.968, *P*<.001). The x-axis indicates the total score for the paper-based JACQLQ, and the y-axis the total score for the app-based JACQLQ.

**Figure 2:**
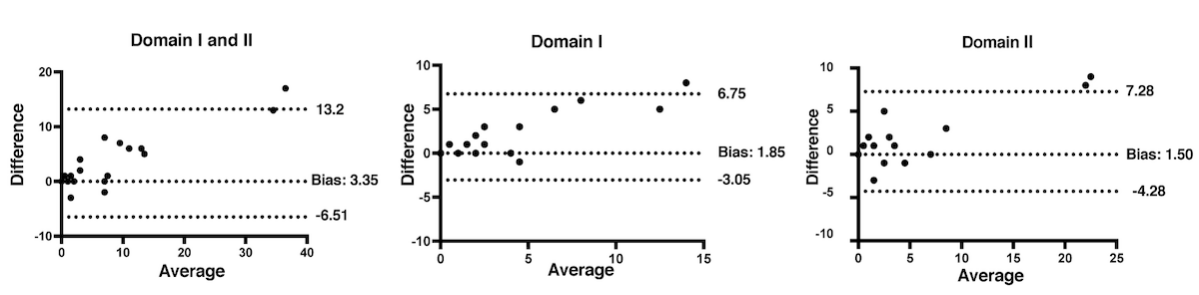
Bland-Altman plot for the paper-based and app-based Japanese Allergic Conjunctival Disease Standard Quality of Life Questionnaires (JACQLQs). The x-axis indicates the average of the 2 methods’ scores, and the y-axis indicates the difference between the 2 methods’ scores. The central line indicates the mean difference (bias) between the scores from the 2 methods, whereas the superior and inferior lines depict the intervals, which include the 95% limits of agreement. Differences between the JACQLQ of Domains I and II, Domain I, and Domain II.

Figure 2 shows the Bland-Altman analysis for the clinical agreement between the paper-based and app-based JACQLQ, revealing a difference (bias) with 95% limits of agreement of 3.35 (–6.51 to 13.2) units for Domains I and II, 1.85 (–3.05 to 6.75) units for Domain I, and 1.50 (–4.28 to 7.28) units for Domain II.

## Discussion

### Principal Results

Hay fever, a highly heterogenous and multifactorial disease, requires personalized assessments to develop effective preventive measures and management strategies. In this study, we examined the reliability and validity of our smartphone app, AllerSearch, regarding collecting data on hay fever symptoms. Our results indicate that the digital administration of the JACQLQ through AllerSearch shows satisfactory reliability and validity metrics; AllerSearch may therefore be an accessible tool for hay fever management. Its accessibility may prove advantageous for screening the undiagnosed population and promoting early, personalized interventions. The COVID-19 pandemic accelerated the breakthrough and subsequent growth of telemedicine and effective self-management. AllerSearch’s ability to assist in the self-management of hay fever, with its extensive reach, aligns well with the aforementioned changing medical paradigm.

Our results show satisfactory internal consistency for most questionnaire items (Cronbach alpha>.70), except for nasal symptoms in the app-based version (Cronbach alpha=.331). Further, there were significant positive correlations between the 2 measurements (Domains I and II: *r*=0.971, *P*<.001; Domain I: *r*=0.920, *P*<.001; and Domain II: *r*=0.968; *P*<.001). These analyses yielded satisfactory results regarding the reliability and validity metrics of the app-based JACQLQ compared to the paper-based version, suggesting the role of ePROs in the future implementation of mHealth.

Our results also indicate that the app-based collection of nasal symptoms showed low internal consistency, which may lead to a discrepancy between nasal and nonnasal symptom assessments. However, nonnasal symptoms, as well as overall symptoms, maintained a high internal consistency, and the low internal consistency observed for nasal symptoms may be attributed to the small sample size in this study. Future efforts to increase power should be pursued to verify or improve on the observed low internal consistency for nasal symptoms.

Traditionally, in-person assessments have not proved very effective in comprehensive evaluations, mostly owing to the low frequency and time constraints of typical outpatient visits [22]. However, as hay fever presents itself as a heterogenous, systemic disease with possible long-term detrimental effects, a holistic evaluation through established questionnaires, such as the JACQLQ, becomes crucial in selecting appropriate treatment regimens. Our findings revealed satisfactory internal consistency and a statistically significant correlation between the paper-based and app-based JACQLQ. It is noteworthy that the Bland-Altman plot analysis on the agreement between the paper-based and app-based JACQLQ resulted in a higher mean (bias) of 3.35 units (95% limits of agreement: –6.51 to 13.2) of the latter compared with the former. We believe that this is owing to a carryover effect stemming from the study design, in which a procedure of the study flow affects another downstream result [23]. We administered the app-based JACQLQ after the paper-based version, and future studies should address the discrepancy through a crossover trial with mixed cohorts on the questionnaire administration order.

Another explanation for the 3-point mean difference between the 2 platforms could be the length and order of the questionnaire items in the app-based version. Demographic and medical history questions preceded the JACQLQ, which might have led to response fatigue [24], a frequently observed phenomenon with survey-type research methodologies. The app-based JACQLQ, by default, positions the scale bar for responses at 0, which may have predisposed fatigued users to quickly answer the JACQLQ items with a low score [25]. This could partially explain the higher score in the paper-based version, as it does not have a “default” score. For further validation of mHealth-driven ePROs and to minimize response fatigue, trials to reduce the number of questionnaire items and reorganize their sequence may be required. Although response fatigue can be addressed, it is practically inevitable; hence, we suggest that the psychological aspects and the resultant discrepancy based on the temporality of the answered items that may have affected the study’s results should be considered. Future studies should also address the interface-led bias and discrepancies between the digital and paper questionnaires, one of which may call for a distinct cutoff score in the digital version for diagnoses and severity assessments. The 3-point mean difference, which does not appear highly relevant from a clinical perspective at this stage, and the consistent correlation between the 2 platforms suggest that ePROs collected through AllerSearch may be valid and feasible for assessing hay fever symptoms and advising on self-management.

### Limitations

This study has several limitations. First, there may be a degree of selection bias stemming from the participants’ demographics, including age and gender. This was also a single-center study, making the selection process prone to selection bias. Further, while there has not been a study, to the best of our knowledge, comparing paper-based and app-based questionnaires, this study had a smaller sample size in comparison to previous studies that investigated discrepancies between digital and paper questionnaires [18,24,26]. Therefore, greater sample sizes are needed for generalization. Lastly, this study did not involve any in-person clinical evaluations on allergic conjunctivitis and did not investigate the correlation of clinical findings with the JACQLQ results obtained through AllerSearch. Therefore, any capability of AllerSearch regarding allergic conjunctivitis diagnosis and screening should not be inferred from our results.

### Conclusions

Our findings indicate that the data collected through the AllerSearch app had good internal consistency, with a Cronbach alpha of >.70 and significant positive correlations between the paper-based and app-based JACQLQ (Domains I and II: *r*=0.971; Domain I: *r*=0.920; and Domain II: *r*=0.968), as an instrument for hay fever symptom management. The mHealth-based PRO enables tailored, longitudinal data-based hay fever management and helps improve patients’ quality of life.

## Abbreviations

e-PRO: electronic patient-related outcome
JACQLQ: Japanese Allergic Conjunctival Disease Standard Quality of Life Questionnaire
mHealth: mobile health
PRO: patient-reported outcome

## Data Availability

All data generated or analyzed during this study are included in this published article.
